# Assessing the Effects of VEGF Releasing Microspheres on the Angiogenic and Foreign Body Response to a 3D Printed Silicone-Based Macroencapsulation Device

**DOI:** 10.3390/pharmaceutics13122077

**Published:** 2021-12-04

**Authors:** Ruth E. Levey, Fergal B. Coulter, Karina C. Scheiner, Stefano Deotti, Scott T. Robinson, Liam McDonough, Thanh T. Nguyen, Rob Steendam, Mark Canney, Robert Wylie, Liam P. Burke, Eimear B. Dolan, Peter Dockery, Helena M. Kelly, Giulio Ghersi, Wim E. Hennink, Robbert J. Kok, Eoin O’Cearbhaill, Garry P. Duffy

**Affiliations:** 1 Discipline of Anatomy & Regenerative Medicine Institute, School of Medicine, College of Medicine, Nursing and Health Sciences National University of Ireland Galway, H91 W5P7 Galway, Ireland; ruth.levey@nuigalway.ie (R.E.L.); scott.t.robinson@gmail.com (S.T.R.); mark.canney@nuigalway.ie (M.C.); robert.wylie@nuigalway.ie (R.W.); peter.dockery@nuigalway.ie (P.D.); 2 UCD Centre for Biomedical Engineering, School of Mechanical and Materials Engineering, University College Dublin, D04 V1W8 Dublin, Ireland; fergal.coulter@mat.ethz.ch (F.B.C.); Stefanodeotti@gmail.com (S.D.); eoin.ocearbhaill@ucd.ie (E.O.); 3 Department of Pharmaceutics, Utrecht Institute of Pharmaceutical Sciences, Utrecht University, Universiteitsweg 99, 3584 CG Utrecht, The Netherlands; karina.scheiner@gmail.com (K.C.S.); w.e.hennink@uu.nl (W.E.H.); r.j.kok@uu.nl (R.J.K.); 4 School of Pharmacy, Royal College of Surgeons in Ireland, D02 YN77 Dublin, Ireland; liammcdonough@rcsi.ie (L.M.); helenakelly@rcsi.ie (H.M.K.); 5 Tissue Engineering Research Group (TERG), Department of Anatomy, Royal College of Surgeons in Ireland (RSCI), D02 YN77 Dublin, Ireland; 6 InnoCore Pharmaceuticals B.V., L.J. Zielstraweg 1, 9713 GX Groningen, The Netherlands; t.nguyen@innocorepharma.com (T.T.N.); r.steendam@innocorepharma.com (R.S.); 7 Discipline of Bacteriology, School of Medicine, National University of Ireland Galway, H91 W5P7 Galway, Ireland; liam.burke@nuigalway.ie; 8 Department of Biomedical Engineering, School of Engineering, College of Science and Engineering, H91 W5P7 Galway, Ireland; eimear.dolan@nuigalway.ie; 9 ABIEL srl, Viale delle Scienze ed.16, 90128 Palermo, Italy; g.ghersi@abielbiotech.com; 10 Dipartimento di Scienze e Tecnologie Biologiche, Chimiche e Farmaceutiche, Università degli Studi di Palermo, 90133 Palermo, Italy

**Keywords:** diabetes, prevascularization, drug delivery, VEGF, medical device, multi-scale porosity, angiogenesis

## Abstract

Macroencapsulation systems have been developed to improve islet cell transplantation but can induce a foreign body response (FBR). The development of neovascularization adjacent to the device is vital for the survival of encapsulated islets and is a limitation for long-term device success. Previously we developed additive manufactured multi-scale porosity implants, which demonstrated a 2.5-fold increase in tissue vascularity and integration surrounding the implant when compared to a non-textured implant. In parallel to this, we have developed poly(ε-caprolactone-PEG-ε-caprolactone)-*b*-poly(L-lactide) multiblock copolymer microspheres containing VEGF, which exhibited continued release of bioactive VEGF for 4-weeks in vitro. In the present study, we describe the next step towards clinical implementation of an islet macroencapsulation device by combining a multi-scale porosity device with VEGF releasing microspheres in a rodent model to assess prevascularization over a 4-week period. An in vivo estimation of vascular volume showed a significant increase in vascularity (* *p* = 0.0132) surrounding the +VEGF vs. −VEGF devices, however, histological assessment of blood vessels per area revealed no significant difference. Further histological analysis revealed significant increases in blood vessel stability and maturity (** *p* = 0.0040) and vessel diameter size (*** *p* = 0.0002) surrounding the +VEGF devices. We also demonstrate that the addition of VEGF microspheres did not cause a heightened FBR. In conclusion, we demonstrate that the combination of VEGF microspheres with our multi-scale porous macroencapsulation device, can encourage the formation of significantly larger, stable, and mature blood vessels without exacerbating the FBR.

## 1. Introduction

The islets of Langerhans are highly metabolic multi-cell structures, which require large quantities of oxygen and glucose to operate normally. Typically, they receive between 5–15% of the pancreatic blood supply, while only accounting for as little as 1–2% of the healthy pancreatic mass [1,2,3]. The combination of a dense capillary network encompassing the islets and the high islet blood-flow rate guarantee that these specialised cells receive adequate oxygen and nutrient supply for survival and function [4]. Islet isolation procedures can often destroy the native islet vascular networks, causing prolonged hypoxic stress, contributing to a 60% loss in transplanted cells within 48 h post-transplantation [5,6]. For these reasons, the development and distribution of blood vessels surrounding macroencapsulation devices is vital for the survival of encapsulated islets and is a limitation for long-term device success.

Within 3 days after transplantation, islet survival and functional capacity are determined, at which time the surrounding graft is largely avascular [7]. An approach to enhance oxygen supply and graft survival is prevascularization, whereby a non-vascularized encapsulation device is implanted days or weeks prior to islet delivery [8,9,10,11,12]. Previous studies by Padera and Colton examining the ideal time course of microarchitecture-driven vascularization demonstrated a time-frame comparable to the typical wound healing cascade of 7–21 days and diminishing by day 329. They found that the number of stable vascular structures plateaued on day 21 and remained unchanged on day 329. This differed from the typical wound healing cascade as the abundance of stable vessels is expected to decline due to regression [13]. Devices which utilize novel polymers or surface topographies to promote vessel formation can significantly enhance neovascularization in this prevascularization period [14,15,16]. We previously examined novel additive manufacturing techniques to tailor multi-scale porosity on the surface of soft tissue implants. This study found that the degree of tissue integration and vascularity in proximity to the implant is shown to increase 2.5-fold with precisely controlled surface structural complexity [17]. This study proposed that the use of this topographical enhancement could promote the tissue integration and vascularization needed for macroencapsulated islet cell viability and efficacy.

Vascularization surrounding devices has been rapidly promoted by the delivery of protein-based growth factors such as Vascular Endothelial Growth Factor (VEGF) to extra-hepatic sites by facilitating the release of angiogenic signals [18,19,20,21,22,23,24,25]. Transplanted islets will eventually release VEGF in response to reduced blood supply, however, premature delivery of VEGF within encapsulation systems has demonstrated improved engraftment and islet efficacy [18,26,27,28]. Our previous in vitro release studies demonstrated that VEGF-releasing microspheres ensure satisfactory VEGF release for 4 weeks, and could prove to be valuable when used as a prevascularization strategy for artificial pancreas implants [29,30].

We propose that by encapsulating VEGF microspheres within multi-scale porosity macroencapsulation devices as a 4-week prevascularization step, we can promote the development of a substantial vascular network surrounding devices. The novelty of this lies in the combinatory approach by using VEGF releasing microspheres, HA gel for both the prevascularization step and intended islet encapsulation, and our novel multi-scale porosity device. Additive manufactured multi-scale porosity has not been achieved to-date for a long term implantable medical grade material silicone, using multi-axis printing techniques as previously described by Coulter et al. This device not only has the potential to provide tissue attachment to the developing surrounding tissue but also through macroscale porosity can anchor the device and act as a scaffold for angiogenesis. To achieve this end, micro-permeable silicone pouches featuring surface macro texture −/+ VEGF microspheres were implanted sub-muscularly in a rodent model for a period of 28 days. In vivo and histological analysis was performed to compare vascularization and foreign body response induced by −/+ VEGF devices.

## 2. Materials and Methods

### 2.1. Macroencapsulation Device and Contents

#### 2.1.1. Device Fabrication

**Figure 1:**
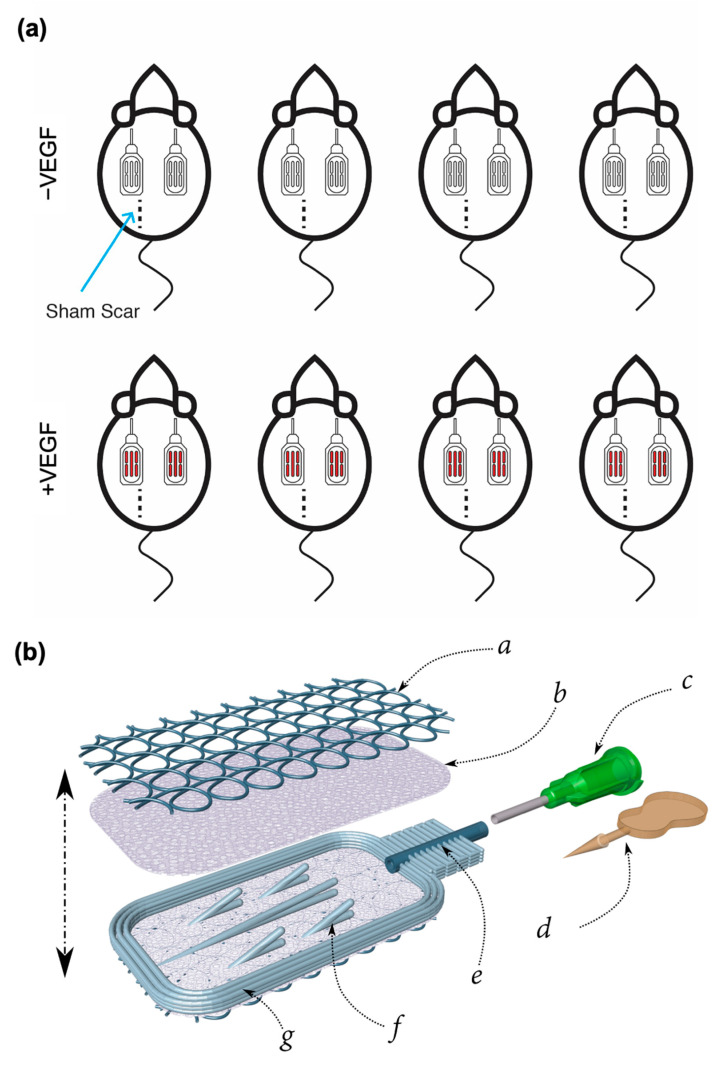
Study design and device overview (**a**) A total of 8 rats were each implanted with 2 textured silicone devices containing a standard HA gel −/+ VEGF microspheres, *n* = 4 per group. (**b**) Schematic of exploded rat-sized (10 × 20 × 1.2 mm) macroencapsulation device. *a*: Non-woven macro-porous pro-angiogenic coiling *b*: Microporous silicone blood barrier membrane *c*: Luer lock input nozzle *d*: Stopper (with break-off tab) *e*: Input tube *f*: Pouch inner structure *g*: Pouch perimeter.

A total of 16 devices of dimensions 10 × 20 × 1.2 mm were fabricated from medical grade silicone (NuSil MED4840) using the combined additive manufacturing processes of Atomising Spray Deposition (ASD) and Direct Ink Writing (DIW) (Figure 1b,c). Devices were composed of two micro-permeable inner membranes (average pore size 6 µm) sandwiched and bonded with an inner support structure. A macro texture was deposited across the outer surface of each membrane in the form of repeating loops (as described in Coulter et al. [17]), and this macro-texture served a dual purpose of reinforcing the membrane and encouraging tissue on-growth.

The membranes were formed by spraying 8 layers of a custom silicone ink on to a hot plate set to 90 °C, and each layer was 8 µm thick. The ink was created by emulsifying a solvent (nHeptane) reduced silicone with a saturated saline solution using a Span 85/Tween 20 surfactant blend. As each layer was deposited, the solvent and water evaporated, letting salt crystals nucleate and interconnect, forming an interpenetrating network of salt and silicone. A fine silicone filament (200 µm in diameter) is deposited in 4 mm width loops upon the membranes, by harnessing the Liquid Rope Coil effect. A total of two more layers of silicone and salt solution are sprayed over the surface. The structure is then cured in an oven at 120 °C.

When fully crosslinked, the membranes are removed from heat, and a 600 µm high inner support structure is printed (with MED4840) on to one membrane. An input tube (800 µm I.D) is placed at one end followed by placing a second membrane on top, to create a pouch. The entire object is returned to the oven for 1 h to fully cure. The salt is washed out of the membranes by sonication in deionised (DI) water for a 24-h period, then sterilised with steam at 121 °C for 15 min before implantation, and each device was filled with 200 μL of Hyaluronic (HA) Acid gel, with the addition or absence of VEGF microspheres, and sealed with a stopper.

#### 2.1.2. Hyaluronic Acid Gel Formulation (VEGF Diluent)

A total of 160 μL of a 1% native HA hydrogel MW: 240–360 kDa (Contipro a.s. Czech Republic) formulation was injected into each device. For −VEGF devices, a 1% *w*/*v* native HA hydrogel was used and for +VEGF devices, a 1% *w*/*v* native HA hydrogel with 12 mg/mL VEGF microspheres (corresponding to a target dose of 150 ng VEGF release per day). HA Hydrogels with VEGF microspheres were formulated using a two-fold concentrated HA hydrogel (~2%), which was then diluted with an equal volume of a 24 mg/mL VEGF microsphere suspension in water. The resulting VEGF/HA hydrogel dispersion was stirred gently with a thin spatula.

#### 2.1.3. VEGF Microsphere Formulation

In order to achieve a sustained local delivery of VEGF for a period of 4 weeks, biodegradable polymeric microspheres loaded with recombinant human VEGF_165_ were used [30]. These VEGF-loaded microspheres were made with (PCL–PEG–PCL)-*b*-(PLLA) multiblock copolymers using a solvent extraction-based membrane emulsification process. VEGF loading within the microspheres was approximately 0.79 wt%, with a loading efficiency of 78%. In the presence of water, the polymer matrix swelled, enabling the diffusion of VEGF from the microspheres. A detailed documentation on the fabrication of these microspheres and their VEGF release properties is reported by Scheiner et al. [29,30].

### 2.2. Sub-Muscular Implantation in Rats

Rodent studies were authorised by the Italian Ministry of Health (Authorization No. 66/2017-PR) and were performed by Abiel Srl (Italy). A total of 8 rats RccHan Wistar (ENVIGO), 150/200 g females, aged 12 weeks, were used during this study. Rats were anesthetized with isofluorane and the implantation sites were shaved and cleaned. A total of three incisions were made in each rat. Of these incisions, two were located to either side of the midline in the thoracic portion of the dorsum. Each incision cut through the dermis to the muscles of the dorsum. Each rat was implanted with 2 devices of the same treatment group (−VEGF or +VEGF) to provide a technical replicate (Figure 1a). Devices were placed sub-muscularly in the cavity following enlargement with a pair of scissors filled and sealed. We have previously demonstrated that sub-muscular implant sites in the anterior abdominal wall meet requirements for size, accessibility, can facilitate longitudinal monitoring of transplants, and can provide nutritive support for cell survival [17,31]. Therefore, a submuscular implantation site was chosen due to its comparability and translatability for our future sites for clinically scaled devices. Each surgical site was closed with 2 or 3 stitches. Devices were also analysed by computerized axial tomography (Capiler CT-Scanner, PerkinElmer). This imaging enabled visualisation of the devices to accurately pinpoint their location and monitor movement. Before sacrifice at 4 weeks, angiogenesis surrounding the devices was evaluated using a non-ionic iodinated contrast agent (Iopamiro^®^ 370). This was performed by cannulation of rat tail vein (cannula 22 G) and continuous perfusion of warmed Iopamiro^®^ 370 at 10 mL/h. Images were acquired with FOV73 and FOV40 cameras. Using the OsiriX Lite program, images were constructed by up-sampling, and used in combination with this software to isolate the complex vessel network surrounding each device and to provide an estimation of vascular volume. Rats were euthanized at 4 weeks. This timeframe was selected as the literature indicated that a constant dose of VEGF for approximately 4 weeks can result in sufficient vascularization in rodent models [32,33]. This timeframe was also previously utilized in our in vitro work for optimizing release of VEGF from the multiblock copolymer microspheres in preparation for preclinical studies [29,30]. Previous studies demonstrating prevascularization strategies using devices alone have also chosen an implantation period of 4 weeks [8,9,11,12].

### 2.3. Tissue Processing

Following euthanasia, the devices were removed en-bloc and fixed in 4% paraformaldehyde overnight. Detailed documentation on fixation, embedding, staining and SEM tissue preparation and imaging were previously reported by Dolan et al. and Coulter et al. [17,34].

### 2.4. Blood Vessel Analysis

The visualisation of blood vessels was facilitated by immunohistological staining of CD31 (ab28364, Abcam, UK), an endothelial cell marker. Using twenty images per sample, a stereological counting technique was used to obtain unbiased estimates of Numerical Density, Length Density and Radial Diffusion Distances [17,34,35,36,37,38]. Each vessel that was counted was also measured for vessel diameter analysis (up to 700 vessels per animal).

In order to further analyse the angiogenic response, the abundance of αSMA (ab5694, Abcam), a cell marker indicative of vessel maturity was quantified. Typically, as blood vessels mature, they become abundant in αSMA expressing cells such as smooth muscle cells, myofibroblasts or pericytes [39,40]. A total of twenty fields of view, chosen randomly, were acquired within each fibrous capsule. αSMA+ vessels within the fibrous capsule were counted using the ImageJ Cell Counter and represented as a percentage of total blood vessels present [17,34].

### 2.5. Fibrous Capsule Analysis

Using Masson’s Trichrome stained tissue sections, morphometric and stereological methods were used to analyse the thickness of the fibrous capsule. Using 4× magnification, ten random images of the fibrous capsule were acquired. Images were captured, compiled into stacks using Image J, and a random offset grid was used to provide test lines. To ensure random measurements were taken of the capsule, the method described here was used [17,34].These measurements were compiled and averaged to calculate the mean fibrous capsule thickness per device. For assessment of fibrous capsule collagen maturity and arrangement, sections were stained with picrosirius red, counterstained with fast green and analysed as previously described [17,34,41,42]. To assess whether the abundance of myofibroblasts was influenced by the presence of VEGF within the devices, the percentage volume of αSMA+ cells was estimated using Image J software as previously described [17,34].

### 2.6. Macrophage Response

The CD68 glycoprotein is commonly used as a pan-macrophage marker secreted by monocytes and tissue macrophages (MCA341r, BIORAD). Using 20 image per sample, the percentage volume of stained macrophages was estimated using a random offset stereological square grid. The number of points landing on CD68+ cells were recorded and represented as a percentage of all points falling within the fibrous capsule.

### 2.7. Statistical Analysis

At least two blind counters were used for analyses. GraphPad Prism was used for statistical analysis. Normality was tested using a Shapiro–Wilk test. For data that were normally distributed, an unpaired *t*-test carried out. For comparing two groups, a one-way or two-way ANOVA with post-hoc Tukey’s multiple comparison was performed. For data that were not normally distributed, a Mann–Whitney U was carried out. Statistical significance was accepted when *p* < 0.05.

## 3. Results

### 3.1. In Vivo Implantation of VEGF Microspheres within a Macroencapsulation Device Increases Neovascularization

**Figure 2:**
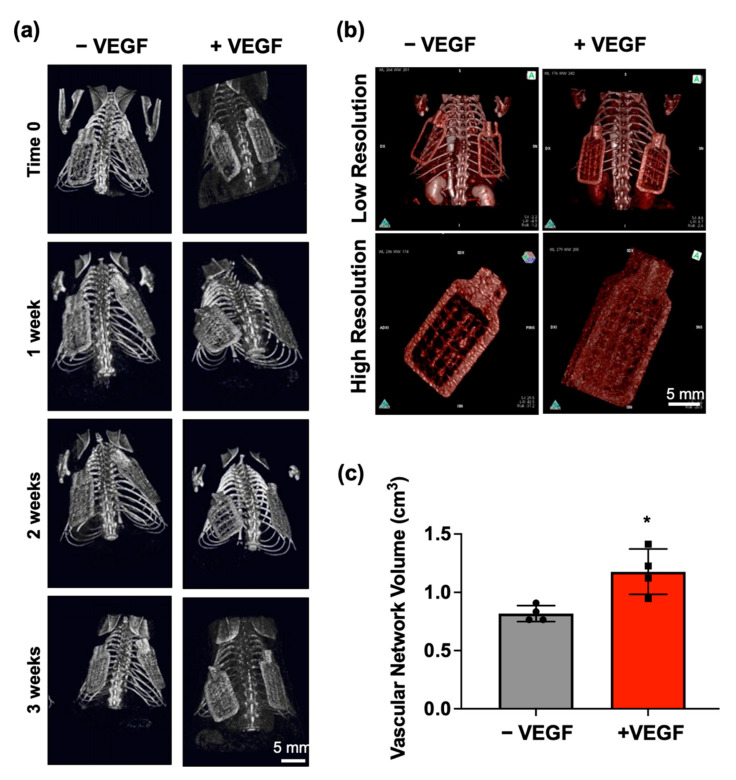
In vivo monitoring of device movement and evaluation of angiogenesis at 4 weeks. (**a**) Weekly representative micro-CT images enabling visualisation of implanted −VEGF and +VEGF macroencapsulation devices to accurately monitor movement over the 4-week period. (**b**) Representative images of Iopamiro^®^ 370 stained −VEGF and +VEGF devices were captured at both low (FOV73 camera) and high resolutions (FOV40 camera). (**c**) Mean volumes of surrounding vascular network estimated using the OsiriX lite program. *n* = 4 per group, data are represented as means ± SD, * *p* < 0.05.

To evaluate feasibility of VEGF microspheres to promote neovascularization in vivo, we first needed to ensure that extreme changes in device position did not occur over the 4-week implantation period, as this could disrupt the newly forming tissue and vasculature surrounding each device. To achieve this end, micro-CT imaging was performed on a weekly basis to enable visualisation of the macroencapsulation devices to accurately pinpoint their location and monitor movement. Over the 4-week implantation period, devices maintained their position and therefore were approved for further analysis as seen in Figure 2a.

Before sacrifice, Iopamiro^®^ 370 staining was performed to permit visualisation of the extensive vessel network surrounding each device and to allow an estimation of vascular volume to be calculated. Qualitative analysis using high resolution imaging showed a greater aggregation and density of vessels surrounding the +VEGF devices when compared to the vessels surrounding −VEGF devices (Figure 2c). Quantitative analysis using the OsiriX Lite software provided an estimation of vascular volume. Normal distribution was observed in both groups and an un-paired *t*-test was performed. A significant increase in vascular network volume (* *p* < 0.0132) was observed in the +VEGF group (Figure 2c).

### 3.2. VEGF Microspheres Increase Vessel Maturity, Stability and Vessel Diameter

**Figure 3:**
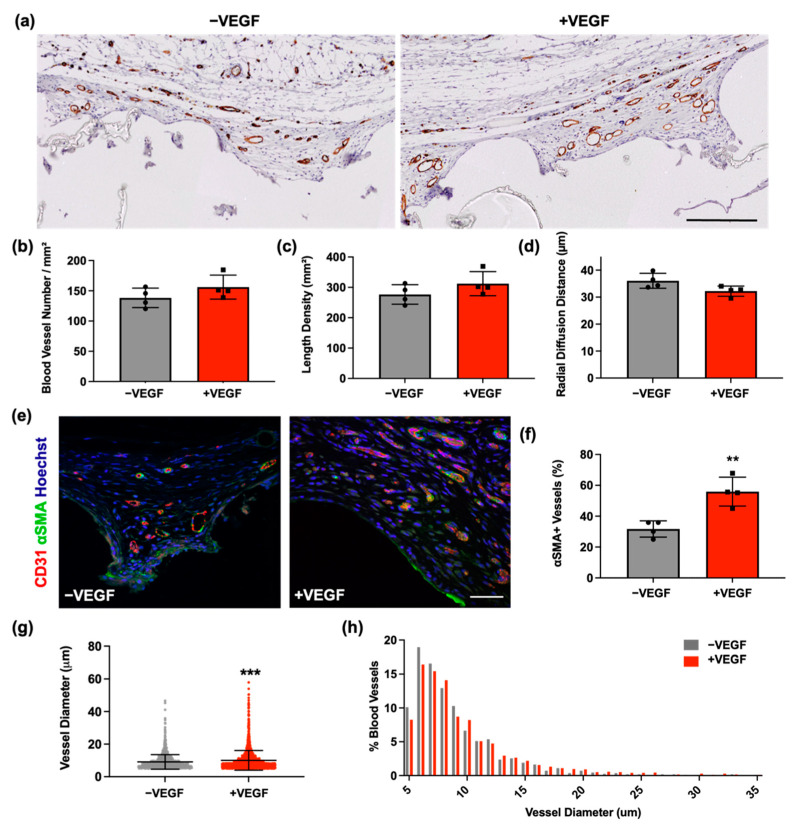
VEGF increases vessel maturity, stability, and vessel diameter. (**a**) Representative images of CD31 staining of vasculature surrounding −VEGF and +VEGF devices. Scale bar = 200 μm. (**b**) Number of blood vessels per mm^2^. (**c**) Length density. (**d**) Radial diffusion distances. (**e**) Representative fluorescent images of αSMA (green) and CD31 (red) staining of fibrous capsules for −VEGF and +VEGF samples. Scale bar = 50 μm. (**f**) Percentage of αSMA+ vessels for analysis of vessel stability and maturity. (**g**) Blood vessel diameters (**h**) Percentage frequency distribution of blood vessel diameters surrounding −VEGF and +VEGF devices. *n* = 4 per group, data are represented as means ± SD, ** *p* < 0.01, *** *p* < 0.001.

To further investigate whether the addition of VEGF microspheres promoted angiogenesis, tissue sections were stained for CD31 and stereological analysis was performed to quantify the blood vessel density surrounding each device (Figure 3a). Normal distribution was observed in all groups and an unpaired *t*-test was performed. Assessment at the tissue–device interface revealed no significant difference in number per unit area (*p* = 0.3288), length density (*p* = 0.3413), and radial diffusion distance (*p* = 0.7811) of blood vessels between −VEGF and +VEGF groups (Figure 3b–d).

Analysis of vessel maturity and stability was estimated by measuring the abundance of αSMA+ blood vessels, a marker indicative of vessel maturity. As blood vessels mature, they become abundant in αSMA expressing cells such as smooth muscle cells, myofibroblasts or pericytes [39,40]. The percentage of αSMA+ vessels was obtained using confocal microscopy (Figure 3e,f). Normal distribution was observed and an unpaired *t*-test was performed. A significantly higher percentage of αSMA+ vessels was observed in the +VEGF group compared to the −VEGF (** *p* = 0.0040).

Subsequently, due to contrasting results, an investigation into blood vessel diameter was carried out. The lumen diameters of >250 CD31+ stained blood vessels were measured per animal and were sampled based on unbiased stereological sampling techniques. Diameters were represented as mean ± SD (Figure 3g). A large variation in data was observed, contributing to a non-normally distributed population. A non-parametric *t*-test was performed with subsequent Mann–Whitney U analysis. Blood vessels surrounding devices containing VEGF microspheres demonstrated a significant increase in diameter when compared to −VEGF devices (*** *p* = 0.0002). As previously performed [43,44], a percentage frequency distribution of vessel diameters was also constructed in order to display the spread of the data based on diameter size (coefficient of variation = 49 vs. 60%) (Figure 3h). The majority of the vessels were between 5–10 µm in diameter. When comparing the size distribution of +VEGF and −VEGF devices, fewer +VEGF blood vessel diameters were found in the 5–10 categories, and a higher proportion of +VEGF vessels were found in the 20–35 µm categories when compared to −VEGF diameters (median = 7.7 vs. 8.2 µm and 90th percentile = 14 vs. 16 µm).

### 3.3. VEGF Microspheres Do Not Cause a Heightened FBR

**Figure 4:**
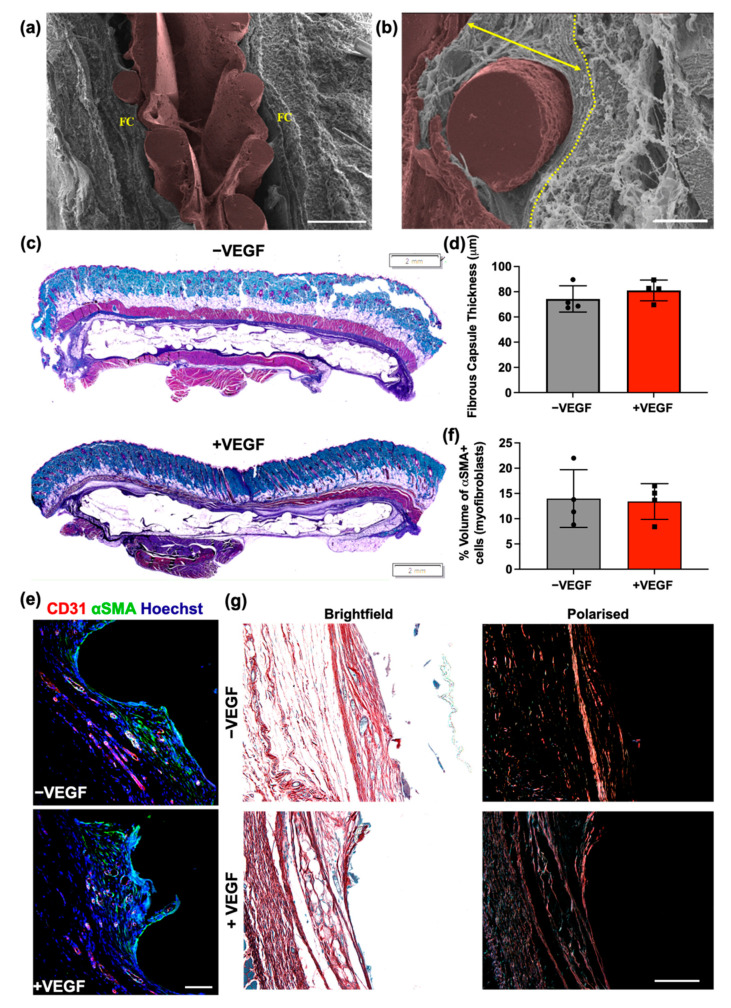
VEGF does not affect the structure and composition of the fibrous capsule. (**a**) Overview of encapsulation device (pseudo-coloured in brown) in-situ with surrounding fibrous capsule (FC). Scale bar = 500 μm. (**b**) Fibrous capsule surrounding rope coil on external surface of +VEGF device. Yellow dotted line marks outer boundary of the fibrous capsule before muscle layer. Arrow demonstrates where an FC measurement would have been taken, perpendicular to the tissue–device interface. Scale bar = 100 μm. (**c**) Representative Masson’s trichrome-stained histological sections of −VEGF and +VEGF groups. Scale bar = 2 mm. (**d**) Mean fibrous capsule thicknesses. (**e**) Representative immunofluorescent images of myofibroblasts within the surrounding fibrous capsule (Hoechst, blue; αSMA, green; CD31, red). (**f**) Percentage volume of αSMA+ cells (myofibroblasts) within the fibrous capsule. (**g**) Representative polarised light microscopy images for analysis of the fibrous capsule and collagen maturity at the tissue–device interface. Scale bar = 100 μm. (Red/Orange = mature collagen; Green/Yellow = immature collagen). *n* = 4 per group, data are represented as means ± SD.

SEM was performed to examine the relationship between the macroencapsulation devices and the surrounding tissue. As shown in a previous study, our multi-scale porosity device results in excellent tissue integration [17]. This finding was also seen in the present study with both −VEGF and +VEGF devices exhibiting an excellent propensity for tissue on-growth and integration into the surrounding tissue as seen in Figure 4a,b.

In order to assess whether the addition of VEGF microspheres affects the degree to which devices became incorporated into surrounding soft tissue, a thickness assessment was performed on the fibrous capsule surrounding both −VEGF and +VEGF groups. Histology was performed on the 4-week explants and fibrous capsule thickness was calculated by measuring thickness of the hyper-dense collagen made visible by Masson’s trichrome stain, shown in Figure 4c,d. Normal distribution was observed in both groups and an unpaired *t*-test was performed. No significant difference was found (*p* = 0.3478) between the treatment groups, indicating that VEGF microspheres do not promote increased fibrous capsule formation.

The percentage of αSMA+ cells within the capsule was estimated using an unbiased point counting technique. αSMA+ cells associated with the CD31+ staining were excluded from this count. Normal distribution was observed in both groups and an unpaired t-test was performed as seen in Figure 4e,f. No significant difference in the presence of myofibroblasts was found (*p* = 0.8685) between the treatment groups, indicating that VEGF microspheres do not promote increased abundance of myofibroblasts within the fibrous capsule. This finding generally correlates with the fibrous capsule thickness results seen in Figure 4d.

Collagen fibres were characteristically birefringent, which was improved with Picrosirius red staining. Polarised light microscopy (PLM) of picrosirius red stained tissue sections was carried out to assess the organization of the collagenous network of the resulting fibrous capsule. Isotropic orientation of collagen deposition with highly organised collagen structure was evident in both treatment groups (Figure 4g). The collagen structure generated in both groups ran parallel to the surface of the device, forming a capsule of organized layers intertwined around the external features of the devices. The majority of the fibres appeared red or orange, indicating the presence of a predominantly mature collagen type I [45,46,47]. Overall, these data suggest that the addition of VEGF microspheres does not influence the devices affinity for integration into the surrounding tissue.

### 3.4. VEGF Does Not Cause a Heightened Macrophage Response

**Figure 5:**
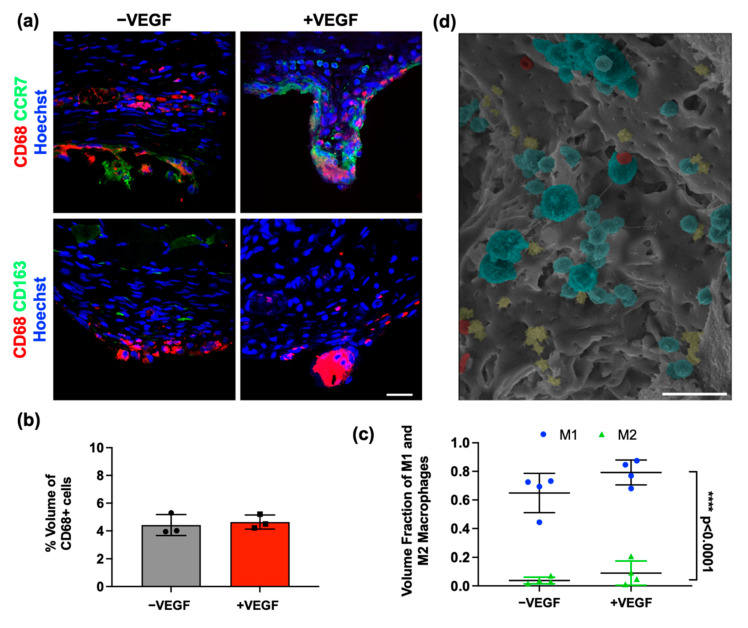
VEGF does not cause a heightened inflammatory response. (**a**) Representative images of CD68 and CCR7 (M1-like) phenotype marker (Hoechst, blue; CCR7, green; CD68, red) and CD68 and CD163 (M2-like) phenotype marker (Hoechst, blue; CD163, green; CD68, red) for both treatment groups. Scale bar = 20 μm. (**b**) Percentage volume of CD68+ (pan-macrophage marker) cells. (**c**) Percentage volume of CCR7+ and CD163+ macrophages. (**d**) SEM image demonstrating an aggregation of cells on the diffusion membrane of the device (macrophages, blue; erythrocytes, red, lymphocytes, yellow). Scale bar = 50 μm. *n* = 4 per group, data are represented as means ± SD, M1 vs. M2 **** *p* < 0.0001.

To establish whether the addition of VEGF microspheres affected the activation and abundance of macrophages in the fibrous capsule, the tissue was stained for a pan-macrophage marker (CD68) (Figure 5a). SEM imaging enabled visualisation of immune cell aggregation on the tissue–device interface (Figure 5d). Percentage volume analysis of CD68+ cells surrounding each device was calculated. Normal distribution was observed, and a parametric unpaired *t*-test was carried out, which revealed no significant difference between groups (*p* = 0.7014) (Figure 5b).

To further analyse the effects of VEGF on the immune response, macrophage polarization was investigated. Sections were co-stained with both CD68/CCR7 and CD68/CD163. The percentage of CCR7+ cells to total CD68+ cells was compared to the percentage of CD163 positive cells to total CD68+ cells using confocal microscopy images (Figure 5a). Multiple *t*-tests were performed, which found no significant differences between M1-like or M2-like phenotypes between −VEGF and +VEGF treatment groups (*p* > 0.13). A significantly higher percentage of CCR7+ macrophages (M1-like) compared to CD163+ macrophages (M2-like) was seen in both treatment groups (**** *p* = 0.0001), suggesting the majority of macrophages present display an M1-like, or pro-inflammatory phenotype (Figure 5c). These data indicate that the release of VEGF from microspheres does not exacerbate the macrophage response and that macrophage populations are consistent across all groups, with a consistent phenotype up to 4 weeks.

## 4. Discussion

In this study we describe a 4-week prevascularization approach, which consisted of VEGF microspheres macroencapsulated within an additive manufactured multi-scale porosity macroencapsulation device, implanted sub-muscularly in a rodent model. It has been established that the successful development of a highly interconnected prevascularized network has the potential to resolve the diffusion limitations of newly seeded and transplanted islet cells, and ultimately reduce the period of cellular reconnection to host vasculature and improve islet viability within the macroencapsulation device [22,23,24]. Without sufficient vascularization, cellular function and device efficacy cannot be maintained.

In vivo analysis of angiogenesis demonstrated a significantly increased (* *p* = 0.0132) density of vessels surrounding the +VEGF devices when compared to the vessels surrounding −VEGF devices. However, no significant difference was observed in the CD31 histological analysis of the number of vessels per area, length density, and radial diffusion distance. These findings were unexpected, contradicting the in vivo angiogenesis analysis and therefore warranted further examination of vascularization. Subsequently, an analysis of vessel stability and maturity was carried out, which demonstrated a significantly higher (** *p* = 0.004) percentage of mature vessels surrounding the VEGF loaded devices. This finding suggested that the addition of VEGF may promote the development of more stable and mature blood vessels. Consequently, an investigation into the diameters of the newly formed vasculature was performed [43,44], as the histological analysis performed did not account for the sizes of the vasculature, which may account for the larger vessel density seen when using in vivo imaging. Blood vessels surrounding the +VEGF devices possessed significantly increased diameter measurements when compared to −VEGF devices (*** *p* = 0.0002). When comparing the size distribution of +VEGF and −VEGF devices, fewer +VEGF blood vessel diameters were found in the 5–10 µm categories with a higher proportion of +VEGF vessels found in the 20–35 µm categories when compared to −VEGF diameters. This increase in vessel diameter and maturity correlates with previous in vitro and in vivo studies, suggesting that VEGF_165_ promotes angiogenic sprouting and may also contribute to defining the lumen diameter [48,49,50]. A previous study using quail embryos demonstrated that VEGF_165_ can stimulate the fusion of blood vessels to create vessels with larger lumens [50,51]. However, Nakatsu et al. found the process to be dose dependant. Lower concentrations of VEGF_165_ promoted growth of long, thinner blood vessels, whereas higher concentrations of VEGF, which correlate with the dosage chosen for the present study [29,30], remarkably enhance the vessel diameter [52]. 

Our findings also correspond with Trivedi et al. [1], who infused VEGF into more traditional PTFE-based devices implanted subcutaneously in a rodent model. This study reported a three-fold increase in blood vessels per field of view adjacent to devices subject to the highest dose, compared to an un-infused control. This PTFE-based device is one of the most successfully vascularized islet macroencapsulation devices under development. Researchers implanted the device for a prevascularization phase of 3 months prior to the introduction of islets. Results of this study showed that the required dose of encapsulated islets was reduced 10-fold in the prevascularized devices when compared to a device and islets which were simultaneously implanted [53].

We have previously shown that additive manufacturing of multi-scale porosity on the surface of soft tissue implants can increase vascularity in proximity over 2-fold [17]. As this optimal device design was used in this study, it is expected that it further promoted blood vessel formation, which is most likely attributed to improved cell attachment, alignment patterns, and ability to support a vascularized network. Previous studies have suggested that novel surface topographies can influence the behaviour of cells, however this response is dependent on the dimensions and the specific morphology of the surface topographies utilized [54,55,56]. Brauker et al. produced a hugely influential paper in this field, which described how membrane pore size has a positive correlation to cell infiltration and an altered foreign body reaction that allowed vascular structures to form in close proximity to the device wall [15]. In a more recent study, greater abundances of blood vessels were formed around a large pore mesh in very close proximity to the immunomodulatory membrane, which should improve mass transfer [57]. Rosengren et al. examined the effects of surface roughness by implanting smooth and textured low-density polyethylene disks and observed that the smooth topography developed a thicker fibrous capsule [58]. Following this hypothesis, Khosravi et al. also demonstrated that a nanotopographical surface significantly increased peri-implant blood vessel density on days 7, 11, and 28 [59].

We further investigated the effect of VEGF microspheres on the fibrous capsule composition and FBR. Both groups showed an excellent propensity to encourage tissue attachment. Thickness measurements of the newly formed hyper-dense collagen deposited around the devices was consistent in both treatment groups. These similarities in tissue integration and fibrous capsule thickness correlated with myofibroblast abundance and collagen maturity results. Macrophage presence surrounding the devices was assessed as a measure of the FBR [60]. The percentage volume and macrophage phenotype were examined at the tissue–device interface and although no significant differences between devices was observed, all devices elicited an FBR. As was found in the previous studies, persistently higher levels of the M1-like phenotype are seen in both −/+ VEGF devices [61,62,63]. These data correlate with the percentage volume data, proving that the addition of VEGF in a prevascularization step does not evoke a significantly enhanced macrophage response and that macrophage populations are consistent across all groups, with a consistent phenotype up to 4 weeks [61,62,63].

Taken together, these data indicated that the addition of VEGF microspheres combined with a multi-scale porosity macroencapsulation device had a statistically increased angiogenic response by promoting increased vessel stability, maturity, and vessel size within a 4-week prevascularization time period. The main limitation of the current study is that we did not demonstrate the efficacy and survival of islets within this macroencapsulation system. Our subsequent preclinical study determines the systems potential by macroencapsulating syngeneic islets in an STZ-induced diabetic rodent model for >8 weeks. Results of this study were very promising, and this manuscript is currently in preparation. The successful development of a highly interconnected pre-vascularized network demonstrated in this study has the potential to resolve the diffusion limitations of newly seeded and transplanted islet cells and ultimately reduce the time of cellular reconnection to host vasculature, improving potential encapsulated islet survival. This novel macroencapsulation device not only improved tissue integration and peri-implant vascularity but also has shown to be retrievable and refillable, which is an important consideration for regulatory purposes [17]. We have previously demonstrated the ability to scale these devices for large animal models, while still allowing low profile, minimally invasive delivery, aligned to common interventional procedures [17,64].

In summary, this device design in combination with growth factors presents an alternative approach to pre-vascularization that may help resolve the diffusion limitations of current devices and simulate the native microarchitecture of the cellular cargo.

## Data Availability

Not applicable.
